# An Account of Some of the Most Important Diseases Peculiar to Women

**Published:** 1848-10

**Authors:** 


*An Account of some of the most important Diseases peculiar to Women.* By Robert Gooch, M. D. *With Illustrations. Second (American) Edition.* 8vo. pp. 322. Edw. Barrington & George D. Haswell. Philadelphia: 1848.

This work may rightly be called one of our medical classics, both from the excellence of the matter and beauty of its style; and yet, as it embraces only a limited range of subjects, it has probably never been read by one-twentieth of the physicians of the United States! "*Multum in parvo,*" is the motto of nearly all. One book, or at most a few volumes, must contain the whole range of medicine, if not of human knowledge. Lately, Dictionaries and Cyclopædias, and now manuals, are all the rage. Even *systems* are hardly tolerated; and as for monographs, few publishers have the temerity to attempt their publication, and, far less, their republication. In the present anxiety for improvement and reform in our profession, we should be very glad to chronicle a reform in this respect. Let physicians be less parsimonious in their purchase of books, and more industrious in reading and studying them, and they will soon lose their passion for digests and manuals, and even systematic treatises, and learn to appreciate good monographs. While the former are too frequently the offspring of a bookseller's spirit for speculation, the latter are almost always the productions of actual observers, describing what they have seen, and announcing the suggestions of their own minds, arising from the objects presented to their immediate observation. The volume before us is of this character. It may be regarded as a collection or series of monographs, on some of the severest, and therefore most interesting, of the diseases to which the human female is liable, from one of the most accomplished medical writers in the English language, whether we regard his natural endowments, his educational advantages, or the extraordinary opportunities he possessed for acquiring information on the subjects upon which he has written. Although we have been familiar with the work for a great portion of our professional life, we can still read it with undiminished interest. The narrations are so clear, the reasoning so lucid, the conclusions so just, and the style altogether is so faultless, that it seems always new to us, and we cannot help thanking the publishers for the real service they have rendered to the profession by the republication of this charming volume. We strongly recommend to every *young* physician, at least, to possess himself of this book of Dr. Gooch, if it be only to learn how to observe—how to think—and how to write what he has seen and thought.

